# Not Only Coping: Resilience and Its Sources from a Thomistic Perspective

**DOI:** 10.1007/s10943-022-01711-5

**Published:** 2022-12-01

**Authors:** Piotr Roszak

**Affiliations:** Department of Christian Philosophy, Nicolaus Copernicus University, Str. Gagarina 37, 87100 Toruń, Poland

**Keywords:** Moral resilience, Fortitude, Power, Weakness, Education, Virtue, Hope

## Abstract

In describing the Christian moral ethos, Thomas Aquinas draws attention to the way in which adversities, trials or afflictions are overcome. This paper analyzes two types of resilience present in Aquinas’s thought as well as their sources and manifestations. The first type, moral resilience, is based on the virtue of fortitude, which governs human behavior in the face of great fear. With regard to the second type of resilience, the focus is on showing how grace contributes to increasing power through weakness. In the concluding section, there are also certain suggestions as to how resilience education could be developed.

## Introduction

In the prologue to his commentary on the First Letter of St. Paul to the Thessalonians, Aquinas refers to the biblical vision of Noah’s ark as it is being raised on the waves of the flood: it does not disintegrate when the waters rise and instead floats upon them (Aquinas, 2012c, 1 Thessalonians, prol.). For Aquinas, the rough waters are an image of the tribulations experienced by the Church as a community––an image in which nothing is quenched or drowned but, through elevation of the mind and spiritual consolation, all is saved. This allegorical image of the Church as an ark, widely developed in the Middle Ages—especially by the exegetes of the School of St. Victor in Paris (Janecki, 2022)—perfectly corresponds to resilience. This contemporary concept describes a person’s ability to overcome difficulties: not yielding to pressure but persevering in the midst of unfavorable conditions and designing oneself into the future (Horvat et al., 2022). It describes situations in which adversity does not lead to a breakdown but brings positive effects and furthers the development of the human person. Interestingly, this means that in such cases, unfavorable circumstances have contributed, to some extent, to a change for the better: a new personal power has been perfected in weakness (Franck, 2021; Terelak, 2021; Ungar & Theron, 2020).

Originally, the term “resilience” was used in materials physics and engineering, where it referred to the strength of a material against deformation and to its ability to return to the previous state. This concept can be exemplified by an elastic “rubber eraser” which is hard to break: it bends on both sides, but it will be not defeated by external force; it resists both deformation and disintegration. Over time, the concept became extensively explored in ecology (Holling, 1973, p. 7), psychology (Manciaux, 2010) or literature (Kazmierczak, 2016), with emphasis being placed on the ability to integrate in spite of the difficulties being experienced. It is from there that the concept has recently moved to moral theology, where it is most often associated with fortitude: the virtue thanks to which a person’s balanced attitude in the face of difficulties (understood very broadly) is configured (Cook & White, 2019; Howard & McKaughan, 2022; Krall, 2020).

## Method

This paper uses a hermeneutic method that attempts not only to understand Aquinas’s ideas in their original context but also to relate them to current discussions and challenges. Therefore, reflections on the virtue of fortitude—which emphasize the importance of enduring evil and point to the sources of this attitude—are juxtaposed with contemporary literature on resilience.

## A Thomistic Vision of Moral Life

As Craig Steven Titus notes, in comparison with the social sciences approach, this resilient attitude is not a simple “coping” with hardship. In the face of difficulties, some people are able to survive while others fall. Understanding these phenomena requires placing them within the framework of the meaning of moral life (Oviedo, 2019; Titus, 2006). The point is to look at the principles of human development and flourishing thanks to which we have a better understanding of the necessity of virtues. At the same time, it is a challenge for our theological considerations on the nature of God and for our understanding of events or situations perceived as difficult that are permitted by God. Aquinas correlates this with God’s goodness, which reaches even demons and the temptations or trials to which they subject humans:

…man’s welfare is disposed by Divine providence in two ways: first of all, directly, when a man is brought unto good and withheld from evil; and this is fittingly done through the good angels. In another way, indirectly, as when anyone assailed is exercised by fighting against opposition. It was fitting for this procuring of man’s welfare to be brought about through the wicked spirits, lest they should cease to be of service in the natural order (Aquinas, 2014, I, q. 64, a. 4 resp.).

This statement of Aquinas offers a framework for perceiving difficulties and recognizing their role in strengthening the human being in good (which, by the way, is the essence of “salvation”: *conservari in bono*). Even mistakes made on this journey are useful, and we should be grateful to those who commit them (Roszak, 2017, p. 32). In this paper, however, we are more interested in resilience as a person’s attitude toward difficulties or, in other words, in the vision of Christian life rather than in God’s perspective.

### In the Face of Difficulties: Are Trials and Temptations an Opportunity for Growth?

The terminology that Aquinas uses with reference to difficulties, from *tribulatio* to *adversitas*, *difficultas* and *infirmitas* to *affligio*, presents us with his anthropological vision: a vision in which, besides man’s good and natural desires, there is also a reality that distracts man from good. Despite its potential dangers, Aquinas ultimately perceives it in a positive light because it indirectly helps in the strengthening of a human being (Boyle, 2014).

The significance of these adversities is presented profoundly in his commentary on the words “My grace is sufficient for you, for My strength is made perfect in weakness” from 2 Corinthians 12:9 (Vlg: *virtus in infirmitate perficitur*). Aquinas observes that weaknesses can contribute to strength in two ways: *materialiter*, when they provide the material for an act of fortitude, but also *ocasionaliter*, when they stimulate a concern for good. Trials show a human being’s vulnerability, provoking a response in the form of humility but also affirming the human integrity and capacity to develop oneself through the *habitus*. On account of their orientation toward exercising human abilities and making people stronger, Aquinas affirms that weaknesses are in some sense useful, which is why God permits them—because in this manner, good can be developed. This is also why they are ordained to the good (*ordinaverat hoc ad bonum*). Interestingly, to support his view, Aquinas offers two examples from ancient military practice and politics: the fact that Scipio Africanus did not destroy Carthage completely and that in the case of the conquest of the Promised Land, God permitted peoples to live there even though He knew that they would fight against Israel later.

Thomas interprets the Pauline idea as an indication of where the source of strength lies and uses it to show that we need grace to gain our full strength. From this perspective, it becomes clear why St. Paul prayed and gave thanks for weaknesses: not for their own sake but for the reason that the power of Christ can appear through them. This is a paradox which Aquinas summarizes using the phrase “fire grows in water” (Aquinas, 2012b, 2 Corinthians, c. 12, l. 3).

Difficult experiences and dangers marked by risks, however, have a positive effect, as evidenced by the very etymology of the word “experience,” which “finds its roots in the practical knowledge, skill or competence (*peritia*) that we draw from a trial or danger (*periculum*) once overcome” (Titus, 2006, p. 209). Not all experiences will result in such competence, however, because they require special help in order to become integrated instead of simply becoming a sequence of individual impressions.

Indeed, the virtue of fortitude is forged in the face of dangers and can be manifested in different ways. As he addresses the question of the achievement of the difficult good (*bonum arduum*), Thomas points to two virtues that prepare the human mind to meet the dangers: *magnanimitas* (or *fiducia*) and *magnificentia*. Their fundamental role is to show how a person can rebuild his or her life after an experience of falling and how risks can be taken when it comes to the greater good. They are responsible for the first aspect of fortitude, which Aquinas refers to as *aggredi* (initiative and attack), whereas the second one, *sustinere* (resistance and endurance), is shaped by *patientia* and *perseverantia* (Aquinas, 2014, II-II, q. 128, a. 1 resp.). Thanks to them, a balance is achieved in human life through which one can strive for good in an orderly manner, according to reason and eternal law. The attainment of these virtues—which are integral parts of fortitude—is crucial to moral life because they prepare a person for perfect action (Huzarek, 2021; Irwin, 2021). However, humans also encounter pitfalls in the form of the weakness of *pusillanimitas*, which manifests itself in uncertainty and timidity (Aquinas, 2014, II-II, q. 133, a. 1 resp.). Nevertheless, the characteristic of *arduum* as the object of the virtue of fortitude*,* as noted by Gauthier and quoted by Titus (2006, p. 289), concern greatness or excellence rather than difficulty: in the Thomistic vocabulary, it is a synonym of *magnum*, *altum*, *elevatum* or *excellens*.

In a situation (very common in pastoral care) where something interferes with the achievement of the desired good, there are two possible ways of helping: explanation of the difficulties or survival among them. In these challenging moments, Aquinas recommends what he calls *firmitas animi*, which belongs to the virtue of fortitude (Aquinas, 2014, I-II, q. 65, a. 1 resp.) and can be observed in many aspects of Christian life, such as pilgrimage (Seryczyńska & Duda, 2021). Therefore, he describes it as “strength of mind in bearing with passing trials” (Aquinas, 2014, II-II, q. 58, a. 8 ad 2)*.* This cardinal virtue allows man not to succumb to the fear that arises when the adversities are great: it makes man’s will resistant and rooted in the good of reason (Aquinas, 2014, II-II, q. 123, a. 4 resp.).

From the theological perspective, temporal adversities (such as *poenae*) are not completely removed by the Passion of Christ or reduced in their intensity because as long as we are on our way to beatitude (*in statu viae*), they often have a stimulating effect on human life, leading to the most important “growth of love”: they may turn out to be a remedy for the worst diseases of the human spirit (Aquinas, 2012a, c. 12, l. 3). When we reach heaven, however, all of them will be removed by the application of the power of Christ’s Passion.

### Flourishing and Resilience

For Thomas Aquinas, moral life is not an automatic process of ethical judgment in which we rely on some external principles, since such an approach would reduce moral theology to simply knowing what is allowed and what is not. How can one react when weakness arises? Many people see living a moral life as not backing away an inch, as being rigid, as not crossing a boundary. However, in human moral action, Thomas sees an orientation toward flourishing which is assisted by virtues, and from the perspective of “happiness,” he builds a vision of Christian morality. This is the first goal of moral action: to achieve *beatitudo*, which has both passive and active aspects (Hankey, 2018). Furthermore, this is the correct perspective for resilience: it is a way to achieve happiness that consists in reaching fulfilment in the intellectual and volitional areas. In other words, it is the blossoming of a human being who is anticipating, through faith, eternal happiness: participation in the nature of God.

In light of all these observations, Christian moral life does not consist in eliminating passions but rather in properly ordering them toward a goal according to reason. In Thomistic anthropology, human emotions such as fear are not to be denied or treated as evil; on the contrary, emotions must be used in a creative and consistent way. Fear is not bad in itself (in fact, it has two faces like a coin: a filial one and an enslaving one) and may have a mobilizing rather than paralyzing effect under certain circumstances. Therefore, in an emotional state, one must not focus solely on what is difficult and must instead grasp one’s tendencies to love, both in *concupiscible* passions (regarding what is good and evil in and of itself) and in *irascible* ones (where the criterion is difficulty).

This is why Titus (2006) claims that the virtue of fortitude seems to correspond to what is nowadays referred to as resilience. Although there is no strictly equivalent term for resilience in Aquinas’s vocabulary, fortitude—mainly in the sense of moral strength in its rational, involuntary and affective manifestations—is what clearly corresponds to resilience as a means of overcoming difficulty. Moreover, every virtue is resilient in some sense because in each virtue, it is necessary to find an optimum, a golden mean between extremes, even if this is not a mathematical middle point but a right measure. In Aquinas’s philosophy, feelings are supposed to be managed in a political rather than despotic manner: they should be moved by reason, not by force.

The object of fortitude is what takes place *in sustinendis et repellendis* (Aquinas, 2014, II-II, q. 123, a. 2 resp.), and it is oriented toward *ad resistendum impugnationibus* (Aquinas, 2014, II-II, q. 123, a. 2 ad 2). Passions can be harnessed to confront and overcome difficulties, although Thomas is aware of how passions affect reason and imagination, reducing attention and concentration on achieving goals. Fear can also contribute to reduced motivation by discouraging a person from acting. At the same time, however, fear can be conducive to action, for “if the fear be moderate, without much disturbance of the reason, it conduces to working well, in so far as it causes a certain solicitude, and makes a man take counsel and work with greater attention” (Aquinas, 2014, I-II, q. 44, a. 4 ad 3). A servant’s fear, to quote Aquinas’s example, can contribute to his greater dedication or attention to work. Such fear can be positive in its impact because it stimulates attention and provokes profound reflection and carefulness.

Furthermore, an aspect which should be important to pastoral theology is that fortitude consists in a certain integration between perception, emotion, reason and will. For Thomas, there is no static perfection in his ethics of virtues. Instead, since virtues are interrelated in a variety of ways (ranging from some virtues being constituents of others to the mutual influence of various virtues due to a *nexus virtutum*), understanding the mechanism of resilience requires taking into account and identifying the beneficial influence of many virtues. Fortitude gives rise to an emotional calm thanks to which attachments are created, and these in turn provide security. Resilience does not indicate pre-existing solutions, instead adapting the means to the situation: being resilient does not mean systematically or continuously evading difficulties or avoiding any spiritual fights but rather confronting evil or adversity when appropriate (Huetter, 2014). In this context, an indication of “when” to act can be achieved thanks to the *recta ratio*.

## Two Types of Resilience

For Aquinas, fortitude does not simply mean standing steadfast; it also needs to have a particular goal. Similarly, two external manifestations of courage may be viewed differently in terms of moral judgment depending on their purpose, intention, understanding and context. In this sense, due to the difference between ethics and moral theology in how they perceive the application of healing grace, it is possible to distinguish two types of resilience: natural (moral) resilience and spiritual (graced) resilience.

### Moral Resilience: Overcoming Difficulties with Fortitude

Moral resilience coincides with the virtue of fortitude in the fact that it involves courageous coping with fear and daring, which leads to the recognition of the essence of fortitude as a virtue that perfects the mind in enduring perils. This means resisting corruption, keeping a firm mind in the face of difficulties and being constant in good (*firmus in bono*). As a result, we can observe three stages in resilience as suggested by C. S. Titus: coping, resisting and constructing. Resilience grows in two situations: when we do not believe in our strength and when we see the power of another being who can help us in our difficulties. Fear decreases when hope increases, so in order to be more resilient, it is necessary to be hopeful, which is the virtue of “possibility” (Titus, 2006, p. 275).

Whenever fear emerges, a person must rectify his or her relationship to what stirs that fear. Importantly, this should be based on realistic cognition because our perceptions often rely on imaginary situations that have immobilized us (Cartagena, 2021, p. 4). The goal is to introduce the kind of order that will not permit us to be distracted from the higher good. This translates into the adherence of the will to the good of reason in the face of the greatest evils and into the ability to choose between goods according to their hierarchy. The hierarchy of acquired goods means that it is sometimes necessary to choose something better. At the same time, a decline in resilience can be provoked by a “weakness of the will,” by the consequences of original sin and by defects, or *akrasia* (Lendman, 2020). In this sense, all efforts to strengthen the will are conducive to resilience.

Natural resilience is characterized not only by the act of resisting but also by the positive effects of a psychological endeavor to build one’s life by engaging emotional dispositions while managing them and, at the same time, overcoming the things that make us weak: vainglory, presumption or meanness. The point is that many virtues not only help us respond to adversities but also contribute to character-building (Szutta, 2021). This is why fortitude should not be perceived only as a “shield” to defend us against evil, since its power is also related to the fact that it helps in attaining the good. For precisely that reason, the source of such moral resilience can be found in one’s environment, that is, family, friends and society, because the goal is to develop relations and protect the common good. Creating such resources in support of a resilient attitude seems to be a great pastoral challenge (Łużyński, 2015; Tykarski, 2019).

Thomas also identifies two more sources of moral resilience: moral experience and knowledge. This is due, in part, to the fact that hope (as a passion) strengthens itself in the experience of accomplishing a difficult good through involvement in long-term projects and through support from others.

In fact, a sign of this kind of resilience would be meekness, which—as a virtue—means harnessing and using one’s energy for a good purpose. According to the etymology of the word, it means taming, that is, organizing man’s volitional and affective spheres. It is a sign of strength and power, not one of submission. Therefore, the context of moral resilience in St. Thomas will point to several sources, both internal and external, building an attitude of resilience. For contemporary pastoral application, it is worth noting some of Aquinas’s recommendations regarding what makes us resilient.

- *Firmus contra maiora* When a person confronts a challenging situation that requires greater involvement or dedication, that person is in many cases able to overcome smaller difficulties as well. This requires good cognitive orientation that helps identify the risk as well as the ability to search for possible responses and the affective motivation to act under difficult circumstances.
- *Building dispositions* for acts of courage through premeditation: This enables one to avoid overestimating the fear and instead realign its size and the corresponding fighting strategy by relying on pre-thought ideas so as not to act under the pressure of time. Thomas identifies several methods: self-knowledge about the origin of fear; self-preparation through imagination, emotion control and active discernment; and gaining an understanding of how fear can affect human life (Borji et al., 2020).
- *Deepening goals* In the face of precarious situations, it helps to deepen the awareness of one’s purpose in life and the significance of love and moral norms so that when something unexpected happens, one can approach it in a new and creative manner (Oleksowicz & Huzarek, 2021).
- *Imagination and example* When an unforeseen situation occurs, fortitude can be bolstered with stories that provide examples of courageous people. Furthermore, resilience can be achieved by spending time in the company of people who are resilient (*per connaturalitatem*). In fact, when St. Thomas talks about natural resilience, he uses the image of a teacher and a student and makes references to the art of building (Huzarek, 2018).

### Resilience and Grace

For Aquinas, grace is a *habitus*: not “something” but rather a supernatural capacity or power thanks to which man is renewed and equipped to achieve goals beyond his nature. In the case of resilience, grace increases through the gift of fortitude and through the infused moral virtue of fortitude. The gift of the Holy Spirit, however, is not a new disposition, like a virtue, but a susceptibility to receiving God’s power. It is like opening man to the fullest extent to the action of grace. Thanks to this gift, we can “keep our eternal perspective and [be grounded] in God” (Titus, 2006, p. 293). Thomas places the infused virtue of fortitude in the context of the love of God and *ea que sunt ad finem* and considers it a source of strength that is capable of overcoming moral weakness. Aquinas explains this as follows:

But the Holy Spirit works this in man, by bringing him to everlasting life, which is the end of all good deeds, and the release from all perils. A certain confidence of this is infused into the mind by the Holy Spirit Who expels any fear of the contrary. It is in this sense that fortitude is reckoned a gift of the Holy Spirit. (Aquinas, 2014, II-II, q. 139, a. 1 resp.)

The gift of fortitude offers the reassurance that all dangers will be overcome, and it does so by diminishing *trepidatio*, that is, confusion, agitation or fear as this term could be translated (Aquinas, 2014, II-II, q. 139, a. 1 ad 1). When this fear is only based on the difficult nature of the adversities, the virtue of fortitude is sufficient. However, when there appears human weakness to resist (*infirmitas hominis ad resistendum*), or even powerlessness, the gift of fortitude takes effect.

The goal is to make man persist in the pursuit of good, but this is often difficult when there is a danger to human life. In order to accomplish this, therefore, we need the support of grace, which is the presence of God (as pointed out in the commentary on the Psalms) “in the middle of the city” so that we do not falter or succumb to evil, so that we are supported by God at the beginning of a good deed. In his analysis of the Book of Psalms, St. Thomas captures these moments of “resilience” and shows how to strengthen them through grace so that they are not broken in the face of difficulties when one is attacked by enemies (Roszak, 2013). This is not about any kind of difficulties but about those that “exceed human abilities” (*excedunt humanam facultatem*), even though the gift shares its name with a virtue. In that respect, Aquinas also mentions the need for the gift of counsel, thanks to which focus on a supernatural good appears (Schuhmann &van der Geugten, 2017).

This leads Aquinas to identify the sacrament of Confirmation as a source of strength in the face of the dangers on the path to holiness, which becomes the “format” of Christian life. In many places, Thomas associates this sacrament with the capacity for resilience: “Man, through this sacrament, becomes strong and steadfast in the confession and proclamation of faith before kings and princes” (Aquinas, 2020, Quodlibet XI, q. 7, a. unicus [7] resp.). In a different context, he also notes that “this sacrament is ordained not only to the sanctification of man in himself, but also to strengthen him in his outward combat” (Aquinas, 2014, III, q. 72, a. 4 ad 3). For that reason, one must be “brought forth, so to say, from the hidden recesses of the Church onto the field of battle” to make a public confession of faith in Christ (Aquinas, 1975, c. 60).

## Limitations of the Study

The reflections on the Thomistic understanding of resilience presented in this paper are based on scholastic terminology which is being adapted to contemporary categories. Therefore, although Aquinas did not think explicitly about resilience, what he expressed in his reflections on the virtue of fortitude is undoubtedly applicable in this context. Building practical and empirical verifications is the next step that will help identify the possible applications of Thomas Aquinas’s theoretical theological concepts to the existence of the contemporary people (García-Alandete, 2022).

## Conclusion

In the introduction to his *Critique of Pure Reason*, Kant quotes an anecdote about a light dove in flight who complains that the air disturbs her from flying. To the dove, it seems that she would be better off without it, but she does not know that she would then be unable to fly at all. In this metaphor, flying is a form of resilience as a positive way of facing difficulties.

In the same vein, Pope Francis (2018, sec. 24) wrote: “Let yourself be renewed by the Spirit, so that this can happen, lest you fail in your precious mission. The Lord will bring it to fulfilment despite your mistakes and missteps, provided that you do not abandon the path of love but remain ever open to his supernatural grace, which purifies and enlightens.”

In view of the above, resilience built on religious experience implies a certain educational program that emphasizes perseverance in pursuit of one’s goals rather than a meticulous focus on failures. The mentality according to which all effort should be focused on “avoiding mistakes” is not conducive to building resilience. Instead, it is necessary to cultivate a culture of argumentation, of emphasizing the “why” of things. Religion focuses people on transcendent goals (Platovnjak, 2017): it shows them that the evil which they experience in their lives is not final, and in doing so, it builds a specific resilience and an ability to cope in difficult circumstances. But this is not merely about “coping,” as noted in the title. On the contrary, this is about persevering in the pursuit of goals and about cultivating fortitude, which—in this context—is not understood as bravado but rather as the persistent pursuit of small goals, breaking down large tasks into smaller steps. The understanding that difficulties are an opportunity for growth and not merely an obstacle, with its rich theological background concerning the fact that God allows evil to exist in the world (Roszak, 2022), is an important point in the educational process that leads to achieving resilience. Aquinas recommends practice, but this is practice supported by the broader intellectual framework that religion offers, one through which a wider worldview is possible. A practical example and means of gaining resilience could be a pilgrimage to sacred places (e.g., the Camino de Santiago), which focuses a person’s attention on the goal and teaches them how to overcome everyday obstacles (Brumec et al., 2022; Mróz, 2016). The aim is not to escape from difficulties (Seryczyńska, 2019) but to take on the challenges in a creative way, which in turns calls for character-building—supported by virtue theory.

As Titus (2006) notes, “resilience findings aid in enhancing virtue theory concerning how humans endure difficulty or suffering, hold firm in a painful struggle, resist self-destructive pressures, wait for the attainment of good, persist until the accomplishment of some goal, and even express sorrow as a virtuous good.”
